# Increased influence of ENSO on Antarctic temperature since the Industrial Era

**DOI:** 10.1038/s41598-019-42499-x

**Published:** 2019-04-12

**Authors:** Waliur Rahaman, Sourav Chatterjee, Tariq Ejaz, Meloth Thamban

**Affiliations:** ESSO-National Centre for Polar & Ocean Research, Goa, India

## Abstract

Under the influence of recent global warming, modulation of frequencies and amplitude of El Niño-Southern Oscillation (ENSO) and its impacts on global climate have become great concerns to the global community. Antarctic climate is sensitive to these changes owing to tropical and Southern Hemispheric (SH) teleconnections. Antarctic surface air temperature (SAT) reconstructed approximately for the past five centuries (~1533 to 1993 CE) based on multiple oxygen isotope (δ^18^O) records of ice cores from East and West Antarctica show dominant oscillations in ENSO and Pacific Decadal Oscillation (PDO) frequency bands. Further, variance of the East Antarctica (EA) temperature record shows significant increasing trend at ENSO band and decreasing trend at PDO band since the industrial era (~1850 CE). This observation is consistent with the earlier report of increasing ENSO activity, reconstructed based on tropical-subtropical tree ring records. ENSO influence in the SH high-latitude is known to be characterized by Pacific South American (PSA) pattern reflected in the atmospheric pressure fields. Our investigation of greenhouse gas (GHG) forced model simulation results show an increasing trend in PSA activity since the industrial era. Thus, we suggest ENSO activity and its influence on Antarctic temperature are increasing in response to increasing radiative GHG forcing since the industrial era.

## Introduction

ENSO is a major source of variability in global precipitation and temperature on 2–8 year time scale. Recent studies have suggested that global climate extremes could be amplified by the combined influence of ENSO and PDO^1–3^, however, depending on their phase relationship. Antarctic climate might be sensitive to these tropical changes due to Southern Hemispheric teleconnections^4^. However, it is not yet well understood that how these changes in tropical oscillations and associated climate extremes influence Antarctic climate through space and time particularly in the backdrop of continuing global warming since the industrial era ~1850 Common Era (CE).

To reconstruct past ENSO behavior and predict its future trend, several attempts have been made based on model simulations and proxy records. Results of long term predictions of ENSO in Coupled Model Intercomparison Project phase-5 (CMIP5) show contradictory results^5,6^. A number of attempts have already been made based on tree ring^7,8^ and coral^9–12^ records to understand decadal to centennial scale variability of ENSO and PDO. These records show good agreement in shorter reconstructions; however, they differ in case of longer records^13^. In addition, there are few important caveats attached to tree ring-based reconstruction such as changes in sample depth, tree size, and chronology—some of which coincide with variance changes and hence, they often misrepresents climate signal^14^. Further, such ENSO reconstructions are mostly from the ENSO regions in tropics/subtropics and therefore may not necessarily reflect changes in ENSO influencing beyond the Niño regions. Though ENSO signals (in temperature and precipitation) are recorded in the Antarctic ice sheet, however, due to non-stationary nature^15^ and poor understanding of transfer functions^4^, it becomes difficult to infer about the changes in ENSO behaviour based on multiple ice core records. In present study, we have reconstructed Antarctic surface air temperature (SAT) for the last five centuries from East and West Antarctica ice core records and investigated temperature signals in ENSO and PDO bands to examine long-term variability in their influence on Antarctic SAT. This long-term reconstruction would enable us to discern their trend during the pre- and post-industrial greenhouse warming and examine their phase relationship.

## Results

**Figure 1:**
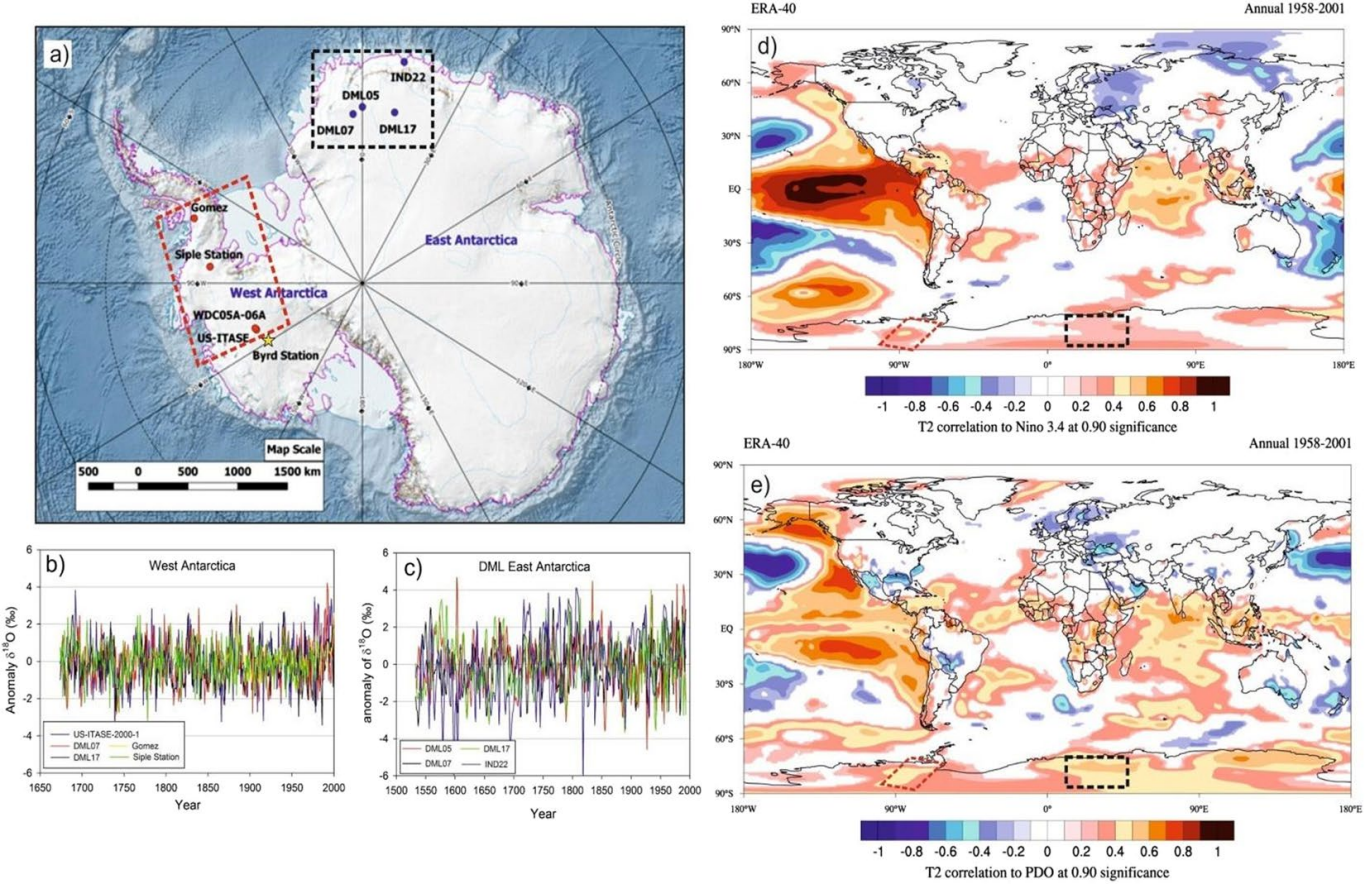
(**a**) Oxygen isotope (δ^18^O) records from East Antarctica: DML05^16^; DML07^21^, IND22^52^, DML17^21^ and from West Antarctica: WDC05A-6A^53^, Siple station^54^, US-ITSE-2000-1^55^ and Gomez^56^. Star represents Byrd station from where data of meteorological parameters are available. (**b**,**c**) δ^18^O anomaly from the West and East Antarctica ice core records. (**d**,**e**) Spatial correlation of ERA 40 Reanalysis surface air temperature with Niño 3.4 index and PDO index. The regions of significant correlation are highlighted in color band. This plot was generated using Climate Reanalyzer (http://cci-reanalyzer.org), Climate Change Institute, University of Maine, USA.

In this study, we used oxygen isotope (δ^18^O) records of ice cores from East and West Antarctica (Fig. 1a, Table S1, and reference therein) that are freely available^16^. These ice core records were chosen based on two criteria; (i) sufficiently longer records with a resolution of minimum one year and (ii) locations of these cores should come under the influence of ENSO and PDO. We found four such records of δ^18^O from East and West Antarctica that are suitable for this purpose (Fig. 1a). Most of these ice core records are obtained from reasonable high accumulation regions (Fig. S1) and therefore provide resolution records at seasonal to annual scales. Uncertainty on these ice cores chronology ranges from ±1 to ±5 years over the past five centuries. Chronology of these ice core records and their uncertainties are discussed in the Supplementary Section S1. The common time interval of the four records ranges from 1533 to 1993 CE and 1674 to 2000 CE for East Antarctica and West Antarctica respectively except Siple ice core (1674 to 1982 CE) from West Antarctica. Therefore, we considered top part (1983–2000) of another record from the Gomez ice core which is adjacent to Siple ice core and stack over the Siple ice core record to make it comparable to the time length of the other three ice core records from West Antarctica (1674 to 2000 CE). By doing so, we could increase the length of the record of common temperature signal extracted from west Antarctic cores and compare top part with the instrumental record of temperature available from the Byrd station (Supplementary Section S2). Spatial correlations of ERA 40 Reanalysis surface air temperature with Niño 3.4 and PDO indices highlights significant (at 90% significance level) positive correlations in these two regions (Fig. 1d,e) and hence, it is expected that the ice core records selected for this study register the signals of ENSO and PDO.

**Figure 2:**
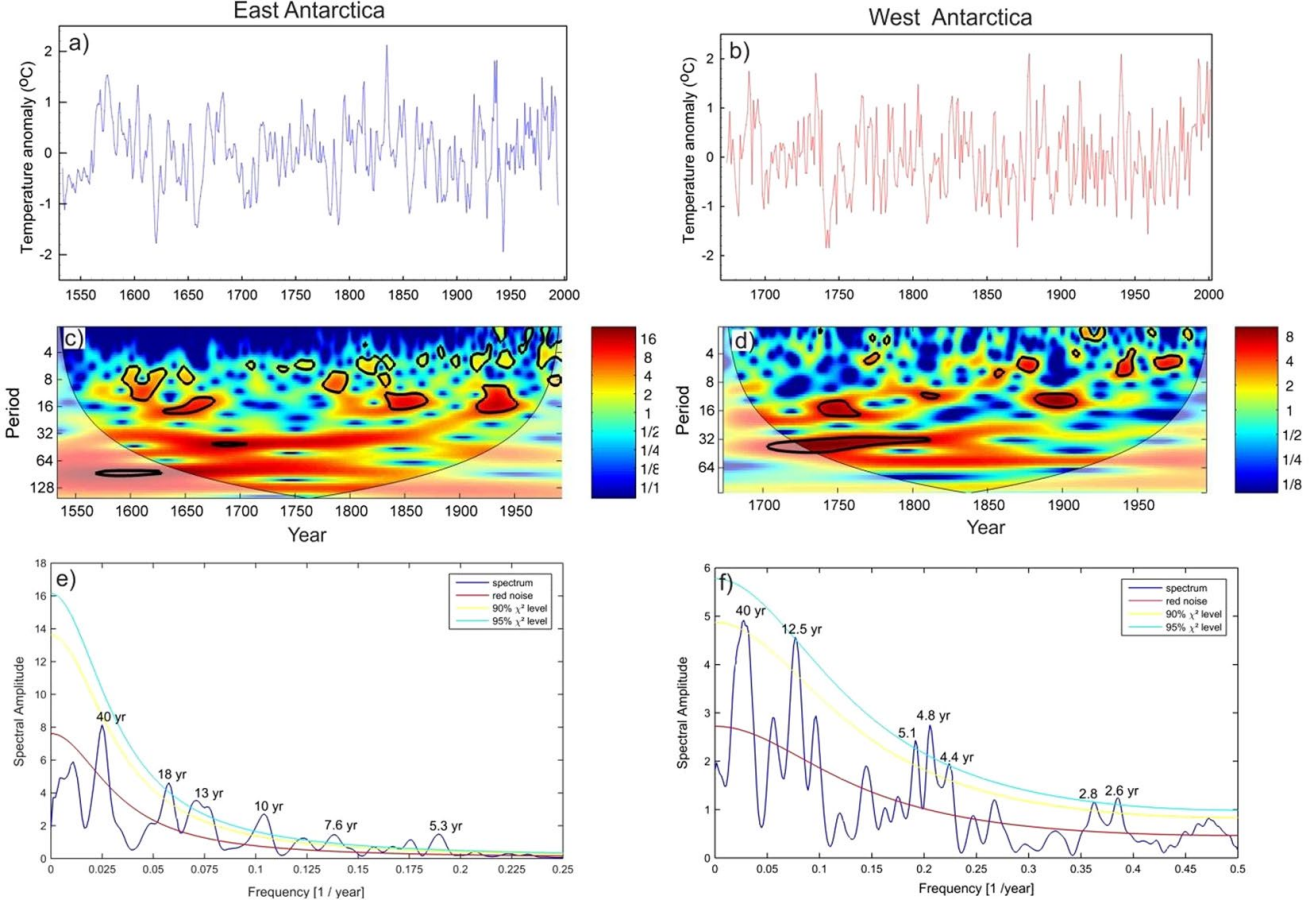
(**a**,**b**) Temperature anomaly time series reconstructed from East and the West Antarctic ice core records. Details of the temperature reconstruction are provided in the Supplementary Information. (**c**,**d**) Morelet wavelet analysis^22^ of the temperature records show how different frequency evolved in frequency- time space. The 5% significance level against red noise is shown as a thick black contour. The thin solid line indicates the ‘cone of influence’, where zero padding has reduced the variance. Yellow and red show increasing spectral power at the corresponding period (colour bar). (**e**,**f**) Spectral analysis of the temperature records using REDFIT software^47^. This shows significant (90% χ^2^ level) periodicities of the temperature records for the East Antarctica: ~5.3, 7.6, 10, 13, 18 and 40 years and west Antarctica: ~2.6, 2.8, 4.4, 4.8, 5.1, 12.5 and 40 years.

**Figure 3:**
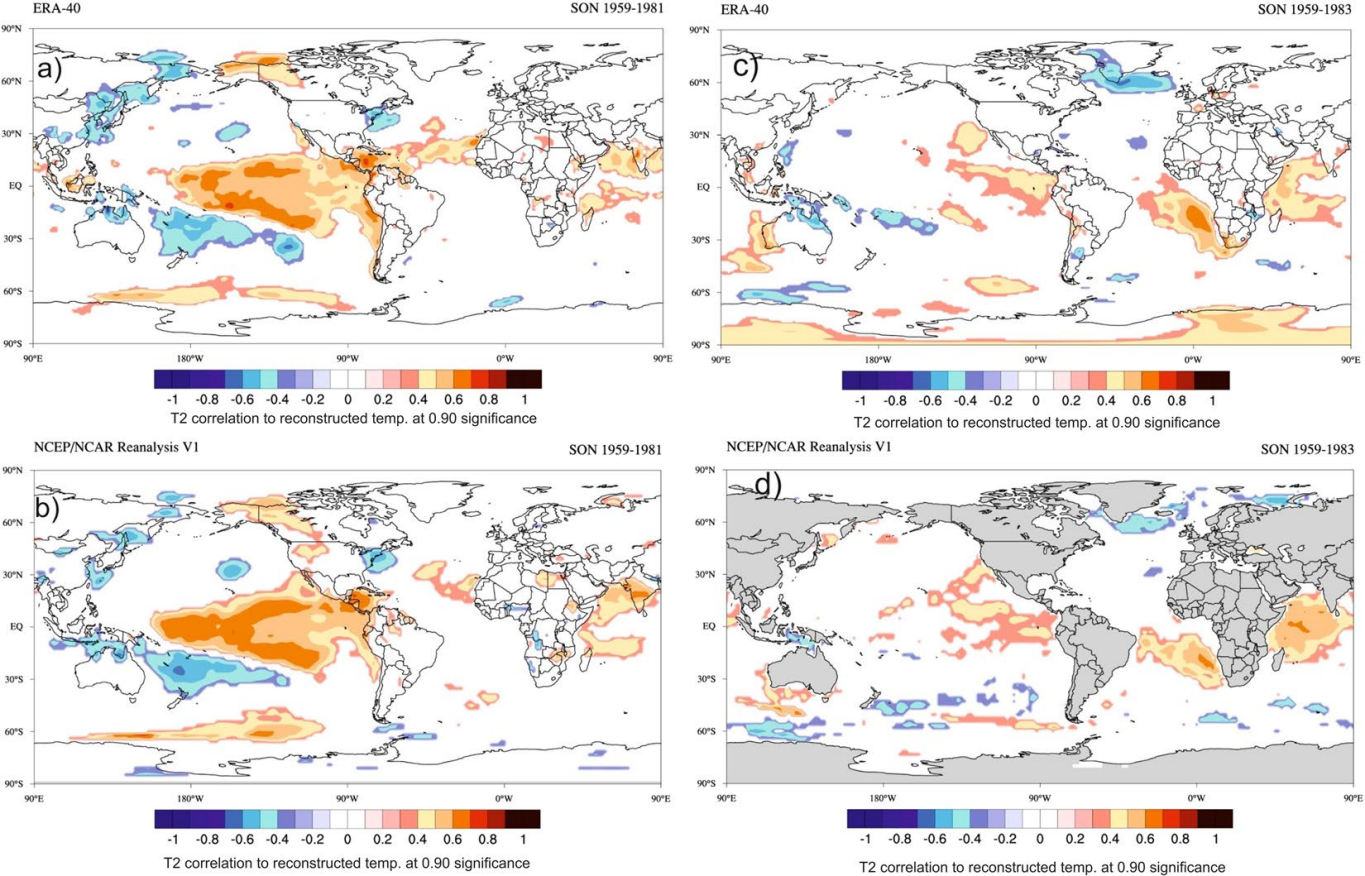
Spatial correlation of reconstructed temperature time series of East Antarctica (1958–1981, 2 years lag) with mean Sept-Oct-Nov (**a**) ERA 40 Reanalysis surface air temperature (at 2 m height) and (**b**) NCEP/NCAR Reanalysis SST. Spatial correlation of reconstructed temperature time series of West Antarctica (1959–1983, 2 years lag) with mean Sept-Oct-Nov (**c**) surface air temperature (at 2 m height) from ERA 40 and (**d**) NCEP/NCAR Reanalysis SST. The regions of significant spatial correlations (at 90% significance level) are highlighted in colour band. This plot was generated using Climate Reanalyzer (http://cci-reanalyzer.org), Climate Change Institute, University of Maine, USA.

**Figure 4:**
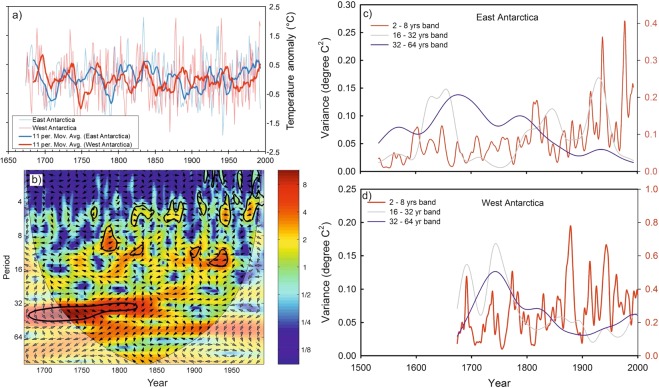
(**a**) Comparison of temperature anomaly records of the East (blue) and West Antarctica (red). Thick solid curves represent corresponding 11-year running average. (**b**) Cross wavelet analysis of the two times series of the East and West Antarctic temperature records. The red color band highlights the common highest power in these two times series. (**c**,**d**) Scale-averaged wavelet power over the 2–8, 16–32, 32–64 yr band which is a measure of the average variance with time^22^. Three curves (red, blue and light gray) represent average variance of the East and West Antarctic temperature at three different frequency bands: 2–8, 16–32 and 32–64 years.

**Figure 5:**
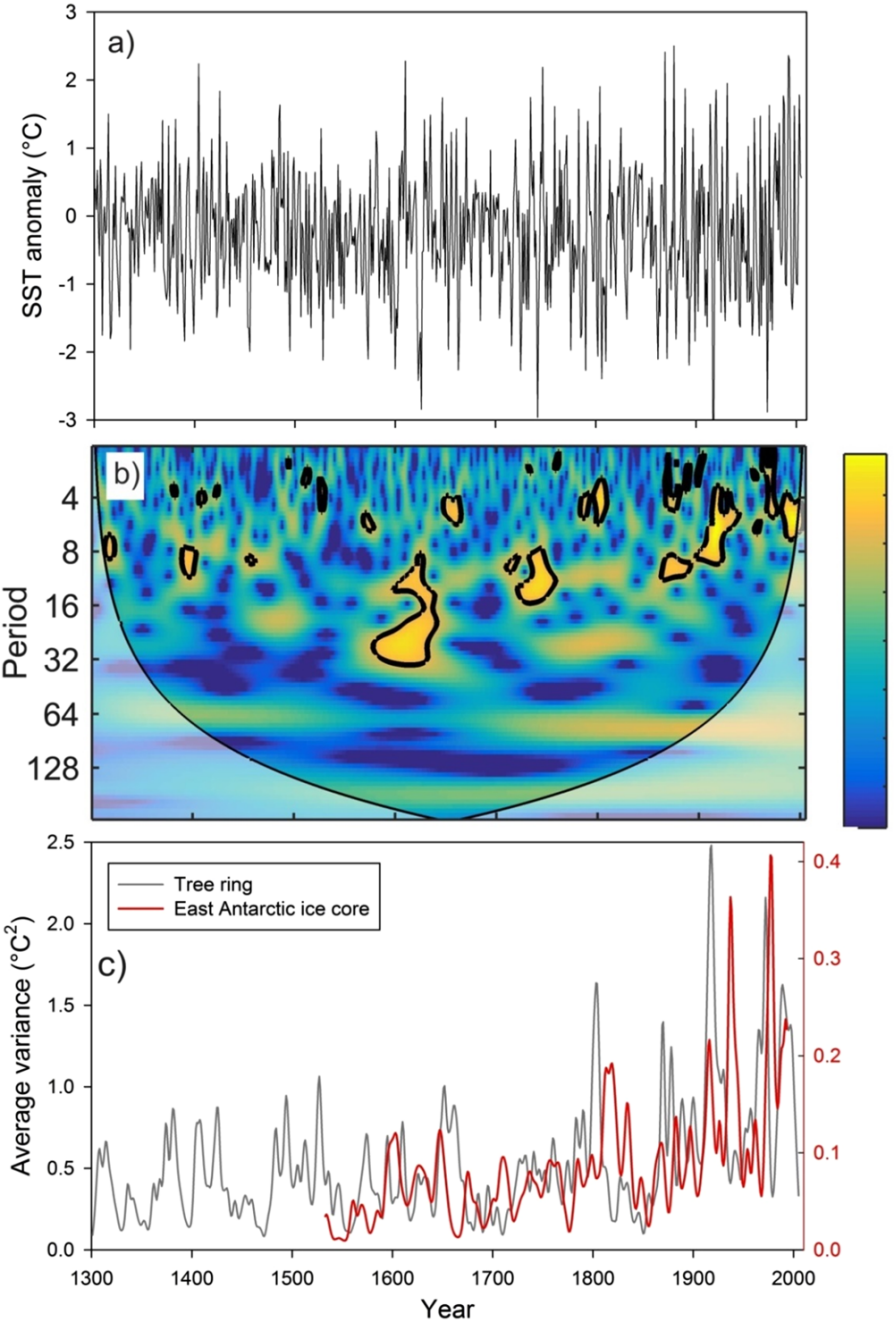
(**a**) Reconstructed Niño 3.4 SSTs over 1301–2005 based on tree ring records from the tropics and mid-latitude^31^. SST anomalies (SSTAs) are relative to the mean of observed SSTs during 1971–2000. (**b**) Continuous wavelet of Niño 3.4 SSTs time series over 1301–2005. This shows significant periodicities at 2–32 years band. (**c**) Average variance of tree ring based SST anomalies (gray curve) and SAT of East Antarctica based on ice core records at ENSO band (2–8 years).

Due to large variability in mean value of δ^18^O records from one to another core, we have compared their anomaly records (Fig. 1b,c). This shows variability in δ^18^O records from East Antarctica is slightly larger than that of West Antarctica. This could be due to the fact that the core locations in the East Antarctica comes under the influence of more cyclonic activity compared to West Antarctica^17^ and receives moisture from both long-range transport and regional/local sources^18^. Though δ^18^O records of Antarctic ice cores have been used extensively to reconstruct past temperature, however, large portion of δ^18^O variability is still unexplained by temperature alone as other factors^19,20^ and post depositional secondary processes are also known to influence δ^18^O values^20^. To examine whether the secondary processes might have played any significant role to alter the climate signal of these ice core records, we compared δ^18^O records with the snow accumulation records available in literature except for two ice cores i.e. Siple station and IND22 (see Supplementary Fig. S1). Overall, snow accumulation in West Antarctic ice cores shows higher accumulation than that of the East Antarctica. All records, obtained from these three ice cores from Dronning Maud Land, East Antarctica, show fairly stable accumulation rates throughout the last nine centuries^21^ (Supplementary Table S1) whereas the west Antarctic ice core sites show relatively more variability. The accumulation rate and δ^18^O show significant correlations in some of the records i.e. DML05 and DML17 (East Antarctica) and US-ITSE-2000-1 (West Antarctica). Therefore, we cannot rule out the possibility of secondary processes that could possible influence δ^18^O-temperature relationship. In order to circumvent this problem, our strategy was to extract maximum temperature signal from multiple ice core records of δ^18^O which would be more reliable and representative to the regions/sectors. In this study, we have reconstructed temperature records separately for the East and West Antarctica based on Principal Component Analysis (PCA) of these ice core records. The first component (PC1) derived from PCA is regarded as temperature which explains maximum variability in the δ^18^O records (See Methods and Supplementary Fig. S2). To confirm whether, PC1 is temperature signal, we have compared with instrumental record available from the Byrd station which shows significant correlations (r = 0.66, n = 45) (Supplementary Fig. S2). In case of East Antarctica, we did not find similar instrumental record in the proximity of the core sites for comparison, therefore, we used reanalysis ERA 40 record of temperature averaged across the region shown in the grid box (76°S to 70°S;12°E to 357°E); they show significant correlations (r = 0.53, n = 35). To translate PC1 time series into temperature record, we employed regression equations to the entire time series of PC1 of the East and West Antarctica (see Supplementary Section S2). Reconstructed annual average temperature anomalies of East and West Antarctica range between −2° to 2 °C (Fig. 2a,b). Wavelet analysis (Morlet wavelet)^22^ decomposes the temperature time series into time and frequency space which shows significant periodicities at different frequency bands ranging from 2–64 years in both East and West Antarctica (Fig. 2c,d). It is noteworthy to observe the increasing trend in high frequency oscillations in 2–8 years band during the past two centuries; however, this is more prominent in East Antarctic record. Spectral analysis of the temperature records shows same periodicities in these two time series. We found significant (90% χ^2^ level) periodicities of ~5.3, 7.6, 10, 13, 18 and 40 years in East Antarctica (Fig. 2e) and ~2.6, 2.8, 4.4, 4.8, 5.1, 12.5 and 40 years (Fig. 2f) in West Antarctica temperature records. The common periodicities of 5.3, 5.1 in ENSO band (2–8 years), ~13 year and ~40 years are observed in both the time series. It is important to identify these periodicities in Antarctic SAT records and find their linkages to climate modes which are known to modulate Antarctic climate. The shorter periodicities in 2–8 years band is likely to be of ENSO signal whereas the longer periodicities in 16–32 and 32–64 years bands are likely to be of PDO signal. In order to confirm whether these periodicities in 2–8 years band in Antarctic SAT records actually represent ENSO signal or not, we performed spatial correlation analysis of mean annual temperature of East Antarctica (1959–1981 CE) and West Antarctica (1959–1983 CE) with ERA 40 surface air temperature (at 2 m height) and NCEP/NCAR Reanalysis SST (Fig. 3) during spring (Sept-Oct-Nov), when ENSO signals are more prominent in the southern high-latitudes^23^. Spatial correlations highlight the regions of significant correlations (at 90% significance level) in color band. The spatial correlation patterns in case of East Antarctica temperature show almost similar to that of the classical ENSO pattern in the Eastern equatorial Pacific (Fig. 3a,b). This indicates response of East Antarctic temperature to the tropical Eastern Pacific temperature variability. However, in case of West Antarctica, it shows weak spatial correlations in the eastern equatorial Pacific (Fig. 3c,d). This could be because of influence of other dominant climate mode i.e. SAM^24^ and Amundsen Sea Low (AML)^25^ which are known to influence more over the West Antarctica and Antarctic Peninsular regions than East Antarctica. In case of East Antarctica, such strong and persistent correlations of temperature records indicate that the non-stationary oscillations in the East Antarctic temperature time series in 2–8 years period represent ENSO variability. The temperature anomalies of both East and West Antarctica show similar extent of variability in both time series (Fig. 4a). However, they do not show any consistent relationship at interannual scale^3^. It is noteworthy to observe that both the records (East and West Antarctica) show discernable increasing trend since ~1950–1993 CE respectively (Fig. 2a,b). The cross wavelet of these two time series highlights the highest common power at 32–64 years bands (Fig. 4b) which is consistent with the observation of common periodicity of ~40 years in the power spectrum analysis (Fig. 2e,f). We also observe that the common periodicity with in-phase relation (vector arrow pointing rightward) at 32–64 years band which is restricted within the time window of ~1650–1850 CE. Further, the cross wavelet also highlights the increasing occurrence of high frequency oscillations at 2–8 years band since ~1850 CE (Fig. 4b). However, discernable increasing trend is observed since the beginning of the 19^th^ century. These evidences clearly indicate shifting in dominant mode of temperature variability from lower to higher frequency band since the 18^th^ century. To quantify relative changes in amplitude of these frequencies, we have performed variance analysis (see methods) for three different frequency bands such as 2–8, 16–32 and 32–64 years bands (Fig. 4c,d). The variance of the temperature records at 2–8 years band shows increasing trend since ~1850 CE, however, dramatic increase is observed since the beginning of the 19^th^ century. The variance in the East Antarctic temperature record at ENSO band shows almost 2–3 times higher in the 19^th^ century over the time interval 1533–1993 CE. This observation is consistent with the earlier reports based on model prediction^26,27^ and multi-proxy records^14,28^. This increase is more prominent and systematic in case of East Antarctic records compared to that of West Antarctica. On contrary, variance at lower frequency band 32–64 years shows monotonic decrease since ~1850 CE (Fig. 4c,d). To confirm whether the temperature signal in this frequency band reflects PDO signal, we have analyzed an independent PDO index reconstructed by D’Arrigo^13,29^. Though multiple reconstructions of the PDO exist but they demonstrate weak coherence prior to the 20^th^ century^30^. We preferred to choose D’Arrigo PDO index over others because it is comparable to our records in terms of resolution and length of the records. Power spectrum analysis of the PDO index shows dominant decadal periodicities of 11, 20, 28 and 50 years (Supplementary, Fig. S3a). Variance analysis of the PDO index at 32–64 years band also shows declining trend since ~1850 CE which is consistent with that of West and East Antarctica (Fig. 4c,d). The observation of decreasing trend in variance of these records since ~1850 CE further confirm that decadal variability of temperature associated with PDO has been declining. However, this is still an open question whether the increase of higher frequency oscillations is at the expense of the decrease in lower frequencies or due to the changes in their phase relationship since the beginning of the industrial era which requires further investigation. Since the timing of increasing trend of ENSO like signals in Antarctic SAT coincides with beginning of the industrial era ~1850 CE pointing towards possible influence of increasing GHG forcing in modulating Antarctic temperature. ENSO reconstruction of the past seven centuries (1301–2005 CE) based on tree ring records from the tropics and mid-latitude have shown increase in ENSO activity in the 20th century in response of continuing global warming^31^. We have analyzed these tree ring based temperature anomaly record (Fig. 5a) and compared with East Antarctic surface temperature. The wavelet and variance analysis of the tree ring based temperature anomaly record shows increasing trend at 2–7 year which is consistent with that of East Antarctic record (Fig. 5b,c). Therefore, concomitant increase in temperature variability at ENSO band since ~1850 CE found in both the records from two spatially distinct regions i.e. low latitude (tropics to mid-latitude) and high latitude (Antarctica) indicate common cause i.e. greenhouse gas (GHGs) induced radiative forcing forcing for such changes. However, it is important to understand how the increase in ENSO activity is reflected in the Antarctic SAT under the greenhouse forcing during the post-industrial era.

**Figure 6:**
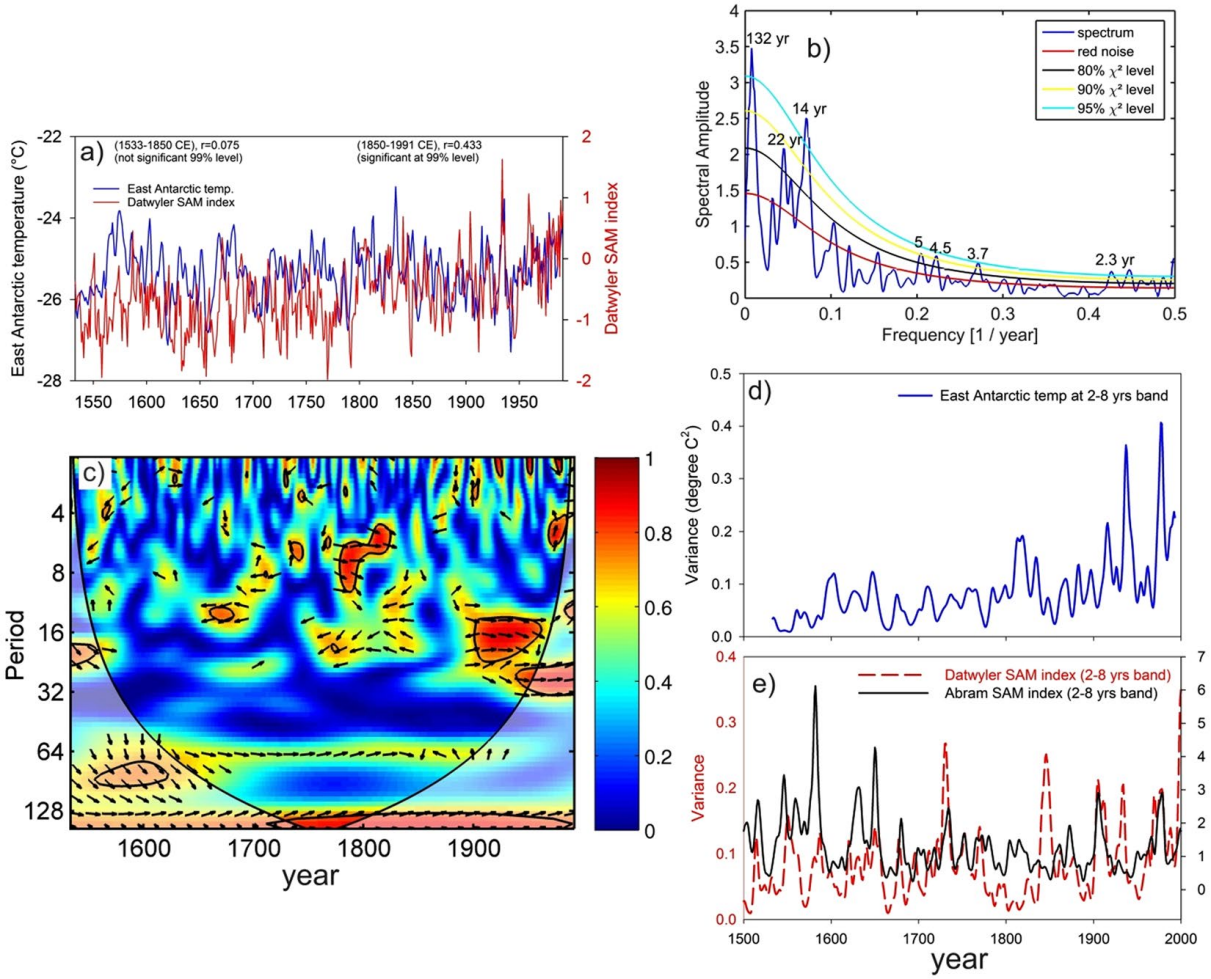
This figure highlights relationship between East Antarctic temperature and SAM at different frequency band. (**a**) Comparison between the East Antarctic time record and Dätwyler SAM index^35^ record. The correlation (r = 0.075) between these time series within the period 1533–1850 CE is not significant (99% significance level) whereas during the interval 1850–1991 CE, r = 0.433 is significant (99% significance level). (**b**) Power spectrum analysis of Dätwyler SAM index^35^ significant periodicities of 132, 22, 14, 5.4, 4.5, 3.7 and 2.3 years at 90% χ2 level. (**c**) Wavelet coherence of two time series i.e. East Antarctic temperature and Dätwyler SAM records. The 5% significance level against red noise is shown as a thick contour. The thin solid line indicates cone of influence. The relative phase relationship is shown as arrows (with in-phase pointing right, anti-phase pointing left, and SAM leading SOI by 90° pointing straight down and vice versa). Scale-averaged wavelet power (average variance) over the 2–8 yr band of (**d**) East Antarctic temperature, (**e**) Dätwyler and Abram^34^ SAM indices.

The core locations selected for this study fall within the Atlantic sector of Antarctica and therefore possibility of Atlantic multi-decadal Oscillations (AMO) cannot be ruled out. However, spatial correlation of ERA-Interim mean annual temperature with AMO index (https://ClimateReanalyzer.org) does not show significant correlation over these regions (Fig. S4). This indicates that AMO might not play direct role in modulating surface temperature of these two regions. Southern Annular Mode (SAM) is one of the primary climate variability in the Southern Hemisphere and known to play dominant role in modulating Antarctic temperature in decadal time scale^32^. Therefore, it is imperative to investigate whether SAM contributed to the increasing trend of the variance in ENSO band. The oxygen isotope used as a temperature proxy generally exhibit inverse relation with SAM as observed in East Antarctic^32^. This relationship was investigated for the shorter scale (since 1960s) and found to be unstable and inconclusive for longer records (1905–2005)^17,33^. We have assessed East Antarctic temperature record with long records of SAM comparable to the temperature time series to investigate its role in influencing East Antarctic temperature. Though SAM beyond the instrumental records (up to 1957) is associated with large uncertainty, but we found two reconstructed SAM records i.e. Abram^34^ and Dätwyler SAM^35^ indices which show overall consistency over the last millennium. For comparison we used the most recently published Dätwyler SAM^35^ index (annual) reconstructed based on correlation plus stationary test (*corr and stat*) of Southern Hemisphere proxy records (Fig. 6a). We calculated Pearson correlation coefficient of two time series in four different time slices i.e. 1900–1991 CE (last century), 1850–1991 CE (post-industrial), 1533–1850 CE (pre-industrial) and 1533–1991 CE (full period), taking into account the effect of their autocorrelation (Table S2, Fig. 6). This shows significant correlation (r = 0.433, n = 141, 99% significance level) during the post-industrial era (1850–1991 CE) whereas it does not show significant correlation (r = 0.075, 95% significance level) during the pre-industrial time slice (1533–1850 CE). This indicates modulation of East Antarctica temperature response to SAM and associated atmospheric circulation patterns during greenhouse warming period. However, it is important to understand at what time scale SAM influences East Antarctic temperature. Power spectrum analysis of Dätwyler SAM index shows significant (95% χ^2^ level) periodicities of 2.3, 3.7, 14 years and 132 years (Fig. 6b). Further, wavelet coherence between the SAM and East Antarctic temperature time series highlight the phase relationship at different frequency bands (Fig. 6c). It is important to note that wavelet coherence does not show any significant phase relation between these two time series at ENSO band (2–8 years). However, we have observed significant coherency of these times series in other frequency bands i.e. in phase relation at 14–32 year band in the last century and 64–128 year band during entire record (Fig. 6c). Further, we have performed variance analysis of Abram and Dätwyler SAM indices in ENSO band and compared with that of East Antarctic temperature record (Fig. 6d,e). It is important to note that variance of SAM records do not show discernable increasing trend in 2–8 years since 1850 CE. Therefore, we don’t find any conclusive evidence which can suggest SAM might influence in increasing variance of East Antarctic temperature at ENSO band.

**Figure 7:**
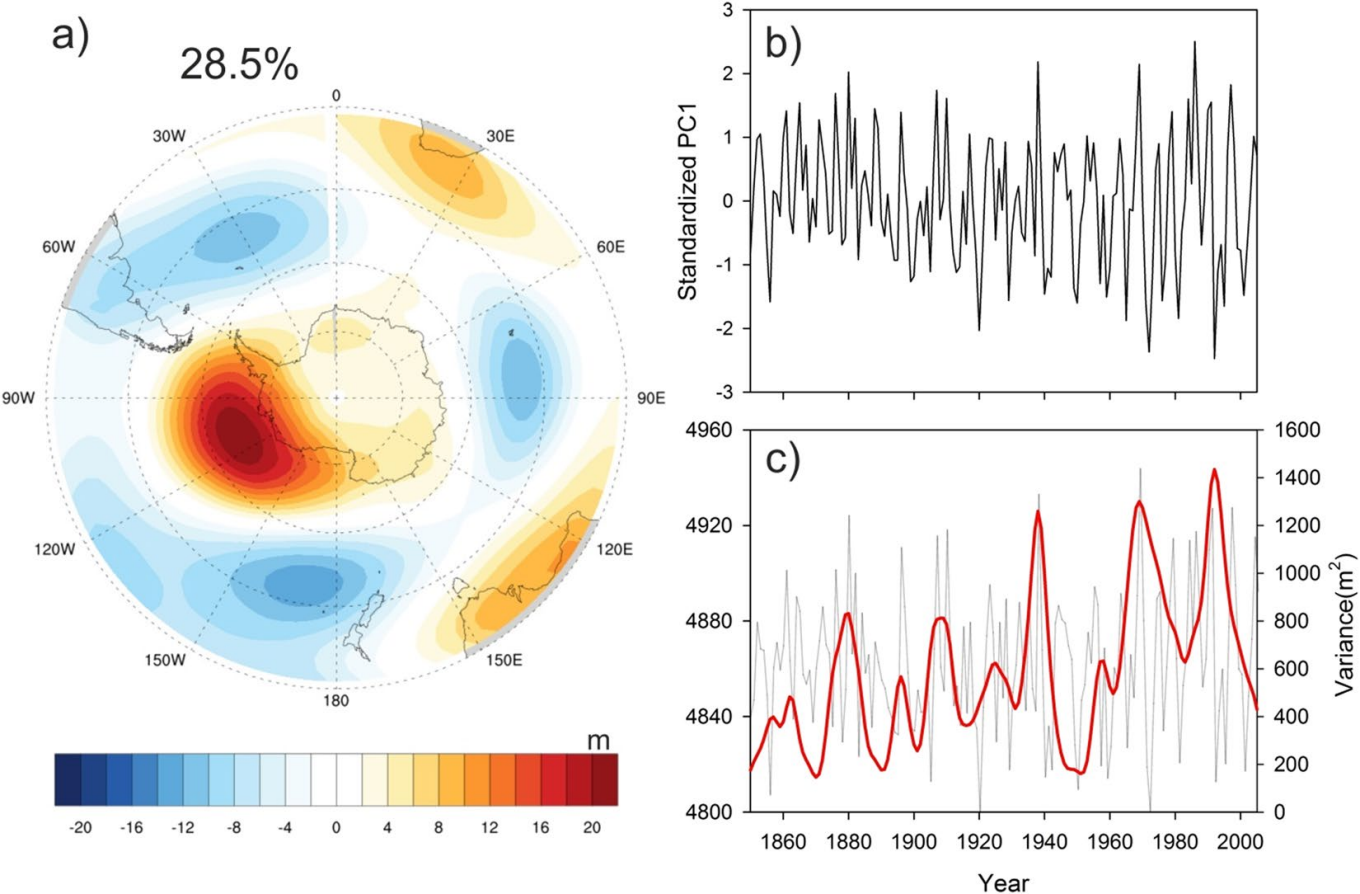
(**a**) First mode of Empirical orthogonal function (EOF), multiplied by the standard deviation of the corresponding principle component, derived from the SON 500 mb geopotential height (z500) over the southern hemisphere (30°S–90°S; 0–360°E) for the period 1850–2005. The pattern depicts the classical Pacific South American (PSA) pattern characterized by a high (low) pressure anomaly over the South Pacific during El Niño (La Niña). EOF1 explains 28.5% of the total variance. This plot was generated using the NCAR Command Language (Version 6.3.0) [Software]. (2015), Boulder, Colorado: UCAR/NCAR/CISL/TDD. http://dx.doi.org/10.5065/D6WD3XH5. (**b**) Standardized Principle component (PC1) associated with the EOF1 pattern in (**a**). (**c**) SON average z500 (m) computed over the region 60°S–75°S, 60°W–120°W (thin grey line) and its average variance at 2–8 year band (red curve) Note that the results are shown for the third ensemble member which shows the distinct amplification of PSA activity. However, similar spatial pattern and its amplification are also found in other two ensemble members of CMSE-LME project (Fig. S5).

The tropical linkage of Antarctic climate comes through variations in SH atmospheric circulation in response to ENSO^4,36^. The evidences of abrupt change in East Antarctic SAT at ENSO band since the industrial era and earlier report of increasing ENSO activity in the 20^th^ century in response of global warming based on tree ring records from the tropics/subtropics indicate a possible amplification of ENSO related variability in SH atmospheric circulation under GHG forcing. Influence of ENSO on the Antarctic climate is known to be associated with the Pacific-South American pattern (PSA) characterized by anomalies in the SH atmospheric pressure fields^23,37,38^. To investigate whether the PSA pattern has been amplified by the post-industrial GHG induced warming, we analyze GHG forced ensemble simulations from the Community Earth System Model-Last Millenium Ensemble (CESM-LME) project (Fig. S5)^39^. The first mode of Empirical Orthogonal function (EOF1) analysis of the SON averaged 500 mb geopotential height (z500) in the SH for the period 1850–2005 explains 28.5% variability and depicts the PSA pattern (Fig. 7a). The time series of EOF1 (PC1) shows increasing interannual variability (Fig. 7b) and variance of z500 over the region shows increasing trend at ENSO band. This indicates increase in PSA pattern activity related to GHG forcing. Thus, we suggest that increasing ENSO activity since the industrial era intensifies its influence on Antarctic surface temperature through stronger responses of SH atmospheric circulation to ENSO under increasing GHG forcing.

### Possible mechanisms for the changing influence of ENSO on Antarctic temperature

**Figure 8:**
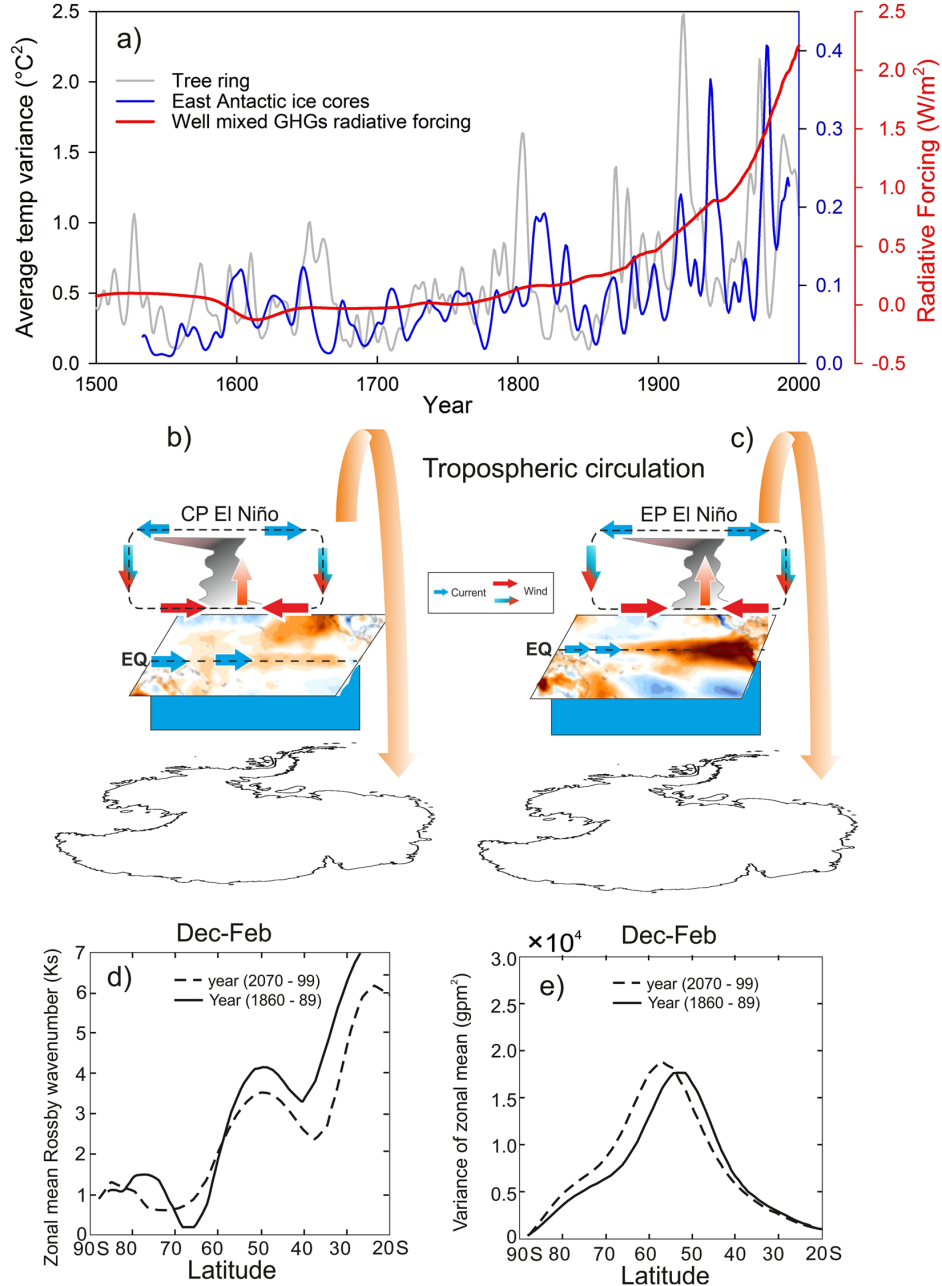
(**a**) Scaled average variance (at ENSO band) in temperature records from the East Antarctic ice core and tree ring are compared with well mixed GHG radiative forcing derived from the PMIP3 model simulations of the last Millennium^40^. This schematic shows longitudinal changes in the SST anomaly during the EP and CP type El Nino (**b**,**c**) and atmospheric teleconnections to Antarctica. (**d**,**e**) This figure shows changes in atmospheric circulation to an enhanced radiative greenhouse gas forcing based on a coupled global climate model (CGCM). This figure is modified from the Brandefelt and Källén^46^. (**d**) Zonal stationary Rossby wavenumber (Ks) and (**e**) variance for summer (Dec–Feb) of the average −30 years for the periods of 1860–89 and 2070–99.

We compare scaled average temperature variance at ENSO band (Antarctic ice core and tree-ring records) with the GHG radiative forcing derived from PMIP3 model simulations of the last millennium^40^ (Fig. 8a). The increasing temperature variance at ENSO band is concomitant with the increase of GHG radiative forcing. The greenhouse gas induced radiative forcing (warming) could change the influence of ENSO on Antarctic temperature in two ways i.e. (i) changes in ENSO properties such as its spatial pattern, amplitude and frequencies and (ii) changes in the ENSO teleconnections to Antarctica. Model simulations corresponding to the time slices and the regions of our interest in the present study would have been more robust approach to find exact reason and unequivocally explain the mechanism for such changes observed in the temperature records which is beyond the scope of the present study. However, considering large number of studies based on the model simulations have already demonstrated how increasing GHG derived radiative forcing has changed the mean oceanic state and ENSO properties, atmospheric circulation and their influence on southern hemisphere temperature. In recent studies, the influence of anthropogenic GHG induced warming on the ENSO type have been examined based on CMIP3 and CMIP5 models^41,42^ and found that the intensity of the central pacific (CP) type ENSO increases steadily from the pre-industrial to the historical simulations and the future projections, but the intensity of the EP ENSO decreases. Changes in ENSO types also affects the teleconnections features in Antarctic temperature as suggested in many studies^43,44^. Here, for an illustrative purpose, we have shown surface temperature response in Antarctica to EP and CP El Niños based two extreme El-Nino 1997/98 (EP type) and 2015/16 (CP type) when both occurred during the positive phase of SAM (Supplementary Fig. S6). We observe almost opposite image in the Antarctic surface air temperature pattern in response to EP and CP type ENSO. Shift in ENSO type can affect the upward propagation of planetary waves into the stratosphere and induce polar temperature^45^ (Fig. 8b,c, this figure is for illustration purpose). Brandefelt and Källén^46^ have investigated the response of the atmospheric circulation to an enhanced radiative greenhouse gas forcing using a transient integration of a coupled global climate model (CGCM). They compared zonal mean and sectorial mean (908W–908E) stationary Rossby wavenumber (Ks) for the periods of year 1860–89 and 2070–99 and observed that the zonal mean MSLP response to an enhanced GHG forcing has changed in the strength and position of this gradient (Fig. 8d,e). This is also observed in increasing PSA activity (Fig. 7c) which is the results of rossby wave trains that generate from tropics.

In conclusion, these evidences suggest that post-industrial increase in radiative greenhouse gas forcing has changed the ENSO properties and mean atmospheric circulation in the southern hemisphere which in turn changed the influence of ENSO on Antarctic temperature.

## Methods

### Reconstruction of past temperature

We used multiple Antarctic ice core records of oxygen isotopes from the recently published global multi-proxy data from the PAGES 2k millennium for this study^16^. Common temperature signal extracted separately for East and West Antarctica based on principal components analysis of oxygen isotope records of ice cores. The first component (PC1) explains maximum variability in δ^18^O records attributed to the surface air temperature change. In order to reconstruct past surface air temperature, we also performed calibration tests by comparing ERA 40 Reanalysis temperature with PC1 (Supplementary Fig. S1) and regression equation was employed to the entire δ^18^O records to reconstruct past temperature anomaly records.

### Power spectrum analysis

A Fortran 90 program (REDFIT)^47^ is used to test if peaks in the spectrum of a time series are significant against the red noise background from a first-order autoregressive (AR1) process. The spectrum of an irregularly spaced time series is determined without the need for interpolation by means of the Lomb-Scargle Fourier transform^48,49^. A Matlab code of this program available online https://www.geo.uni-bremen.de/geomod/staff/mschulz/#software is used to determine the significant periodicities against the red noise.

### Wavelet analysis

The wavelet transform can be used to analyze time series that contain nonstationary power at many different frequencies. We use Morlet wavelet to decompose the time series into time-frequency space which enable us to identify the modes of variability and how those modes vary with time^22^. Statistical significance was estimated against a red noise model. For analysis of the covariance of two time series we used cross wavelet which highlights the common highest power in two time series^50^. Statistical significance is estimated against a red noise model. This wavelet analysis was performed using Matlab code available http://grinsted.github.io/wavelet-coherence/.

### Scaled average variance analysis

To examine fluctuations in power over a range of scales (a band), we used scale-averaged wavelet power which is a time series of the average variance in a certain band. Thus, the scale-averaged wavelet power can be used to examine modulation of one time series by another, or modulation of one frequency by another within the same time series. We have performed this analysis using online Matlab codes http://paos.colorado.edu/research/wavelets/.

### PSA and EOF analysis

Investigations of changes in SH atmospheric circulation in response to ENSO are performed using ensemble simulation with GHG forcing from the CESM-LME project (http://www.cesm.ucar.edu/projects/community-projects/LME/). Three ensemble members are available under this project with transient GHG forcing for the period 850–2005 CE. Since the changes in GHG before 1850 are small and due to natural causes we only investigate the period after 1850 when the GHG increases constantly. The PSA pattern is obtained through EOF analysis of the SON average 500 mb geopotential height in the Southern Hemisphere (30°S–90°S; 0°–360°E) for the period 1850–2005. Before applying EOF analysis the data is weighted with square root of cosine of its latitude. While all three ensemble members shows the similar PSA pattern in EOF analysis, only third ensemble member is used in this study as the increase in PSA activity, as discussed in the text, is most prominent in this member than other two.

### Pearson correlation analysis

Pearson correlation coefficients (r^2^) of two time series at different time slices were calculated, taking into account the effect of their autocorrelation using online available MATLAB codes https://oxlel.zoo.ox.ac.uk/resources/reconstats. Details of the mathematical function and the codes are described elsewhere^51^.

## Supplementary information

Suplimentary information
