# Supplementary material for: Increased influence of ENSO on Antarctic temperature since the Industrial Era
Source: Sci Rep. 2019 Apr 12;9:6006. doi: 10.1038/s41598-019-42499-x (PMC6461609; doi:10.1038/s41598-019-42499-x)
Supplement: Supplementary file 1 — Suplimentary information [file 41598_2019_42499_MOESM1_ESM.docx]

**Supplementary material**

**Increased influence of ENSO on Antarctic temperature since the Industrial Era**

Waliur Rahaman, Sourav Chatterjee, Tariq Ejaz & Meloth Thamban

| **Section** | **Pages** | | |
| --- | --- | --- | --- |
| S1. Chronology of the ice core records and their uncertainty | **1-2** | | |
| S2: Temperature reconstruction from δ^18^O records of Antarctic ice cores | | **5-6** | |
| Table-S1: Details of the ice core records | | **2** | |
| Table-S2 | | **7** | |
| Figure-S1: Accumulation and δ^18^O of East and West ice core records. | | **4** | |
| Figure-S2: Extraction of temperature signals from the oxygen isotopes records of multiple ice cores | | | **6** |
| Figure-S3: Spectrum and variance analysis of the reconstructed temperature of East Antarctica and PDO index | | **7** | |
| Figure-S4: S4 Spatial correlations of ERA-Interim Reanalysis mean annual temperature with Atlantic Multi-decadal Oscillations (AMO) index. | | **8** | |
| Figure-S5: Model run of greenhouse forced Ensembled Empirical orthogonal functions of SON averaged 500mb geopotential height | | **9** | |
| Figure-S6: Fig. S6 Longitudinal changes in SST anomaly during (a) EP type El-Niño (1997/98, Dec-Feb) and CP type El-Nino (2015/16, Dec-Feb). | | **10** | |
| References cited | | **11-13** | |

Table –S1: Details of the ice core records

|  |  |  |  |  |  |  | |  |
| --- | --- | --- | --- | --- | --- | --- | --- | --- |
| **Regions** | **Core Name** | **Latitude** | **Longitude** | **Time range** | **Resolution** | **Average accumulation** | **References** | |
|  |  | °S | °E | Years | Years | kg m^-2^ yr^-1^ |  | |
| East Antarctica | DML05 | 75 | 0 | 166 - 1995 | 1 | 61 ± 12 | 1 | |
|  | DML07 | 75.6 | 3.4 | 1000 - 1994 | 1 | 62 ± 12 | 2 | |
|  | DML17 | 75.2 | 6.5 | 1000 - 1996 | 1 | 45 ± 11 | 2 | |
|  | IND-22 | 70.1 | 11.5 | 1533 - 1993 | 1 | 170 | 3 | |
|  |  |  |  |  |  |  |  | |
| West Antarctica | US-ITSE-2000-1 | 79.4 | 111.24 | 1673 - 2001 | 0.02 - 0.1 | 218 ± 46 | 5 | |
|  | Siple station | 75.9 | 84.3 | 1417 - 1982 | 1 | - | 8 | |
|  | WDC05A | 79.5 | 112.1 | 786 - 2004 | 0.05 - 0.1 | 199 ± 35 | 6 | |
|  | WDC06A | 79.5 | 112.1 | 50 BC - 2006 | 0.05 - 0.1 | 199 ± 35 | 6 | |
|  | Gomez | 73.6 | -70.4 | 1858-2006 | 0.05 - 0.1 | 703 ± 196 | 10 | |
|  |  |  |  |  |  | |  |  |

**Section S1: Chronology of the ice core records and their uncertainty**

The chronology of three ice cores (DML05[^1^](#_ENREF_1), DML07[^2^](#_ENREF_2) and DML17[^2^](#_ENREF_2)) from the Droning Maud Land, East Antarctica have been discussed extensively in earlier publication[^2^](#_ENREF_2). Identification and annual counting of the layers were performed based on the seasonal signals of major ions together with well-known historic volcanic layers. Uncertainty on the chronologies for these cores were ascertained based on several independent checks, is about ±5 years over the past nine century records[^2^](#_ENREF_2). The chronology of another core IND22/B4 ice core was obtained from volcanic stratigraphy using the non-sea-salt sulphate (nssSO_4_^2−^) records[^3^](#_ENREF_3), and later was further confirmed by tephra records[^4^](#_ENREF_4). The known volcanic events reported in the last ~450 years and its corresponding nssSO_4_^2−^ show good agreement with an uncertainty of about ±5 years.

The US ITASE 2000-1[^5^](#_ENREF_5) core from West Antarctica was dated by annual-layer counting, primarily through the identification of summer peaks in non-sea-salt sulfate (nssSO_4_ ^2–^) concentration[^6^](#_ENREF_6). Absolute dating accuracy was better than ±2 years and relative dating accuracy was better than 1 year was demonstrated based on the identification of multiple volcanic marker horizons. The chronology of the two ice cores from the West Antarctica i.e. WAIS Divide ice core (WDC05A[^6^](#_ENREF_6), WDC06A[^6^](#_ENREF_6)) were determined by multiple methods including age of the gases (CO_2_ and CH_4_)[^7^](#_ENREF_7). The estimated uncertainty of the chronology is about ± 10 years in the last two thousand years record. The chronology of the Siple[^8^](#_ENREF_8) station ice core was determined by gas chronology with an accuracy of approximately ±2 years[^9^](#_ENREF_9). The temporal length of the Gomez[^10^](#_ENREF_10) core is 152 years, encompassing 1855 – 2006 and the estimated uncertainty in the dating is ±1 year[^10^](#_ENREF_10). Overall uncertainty discussed here shows large range with maximum uncertainty up to ±10 years, however, depend on the temporal length. The temporal length of our targeted period for this study was about the last five centuries, for which the uncertainty is expected to be better than ±5 years.


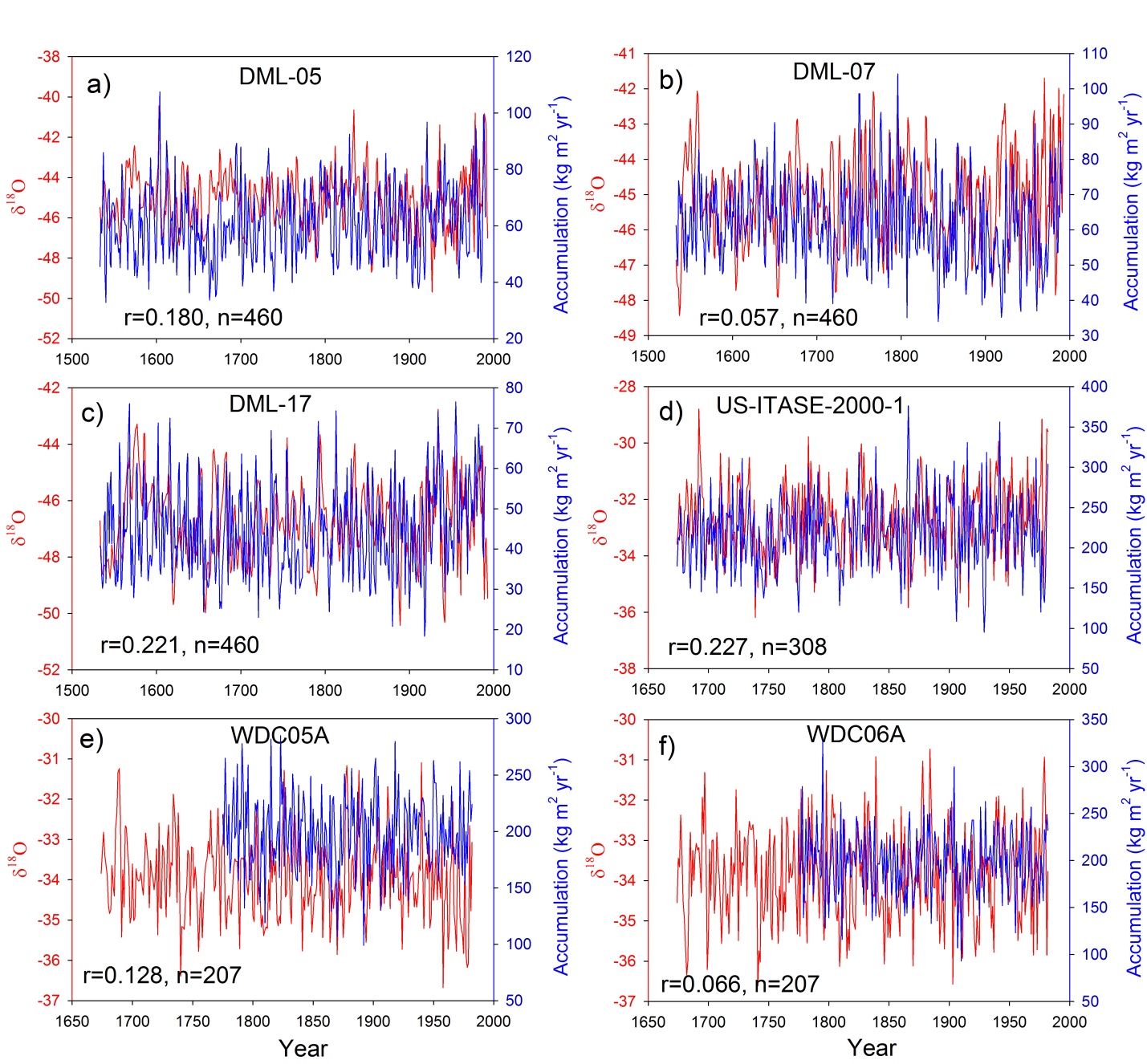


Fig. S1 Accumulation and δ^18^O of East and West ice core records. r and n represent correlation coefficient and number of samples respectively in Pearson’s correlation between δ^18^O and accumulation.

**Section S2: Temperature reconstruction from δ^18^O records of Antarctic ice cores**

We have reconstructed temperature anomaly based on the multiple ice core records δ^18^O from the East and West Antarctica. Though the δ^18^O records is related to surface air temperature, however, they can influenced by the other factors[^11^](#_ENREF_11). Therefore, in order to extract maximum signal of the temperature, we have performed principal component analysis (PCA) and the first principal component (PC1) is expected to be the temperature signal which explains maximum temperature variability in δ^18^O records. However, to confirm, we have compared PC1 of the east Antarctica and west Antarctica separately with temperature records of the Reanalysis ERA 40 and instrumental record from the Byrd station respectively (Fig. 1). ERA40 temperature record represents averaged temperature across the region (76°S-70°S; 12°E-357°E) shown in the grid box (Fig. 1). In case of the west Antarctica, we have compared PC1 (west Antarctica) with the instrumental record of temperature available from the Byrd station located in the proximity of the core sites which is more accurate than the Reanalysis ERA 40 data as reflected in the coefficient of Pearson correlations (East Antarctica: r= 0.53, n=35 and West Antarctica: r=0.66, n=45) (Fig. S2). Subsequently, we have employed these regression equations to convert PC1 records in terms of temperature. The uncertainties associated with slopes of the regression equations, Reanalysis data ERA40 and instrumental record would be propagated to the reconstructed temperature records. We could not constrain the uncertainty associated with the final temperature reconstruction due to limited knowledge on the error associated with their sources. Therefore, we used these temperature records to investigate temperature variability pattern and their link to known climate modes (e.g. ENSO, PDO and SAM) instead of using them to infer about temperature changes in absolute scale.


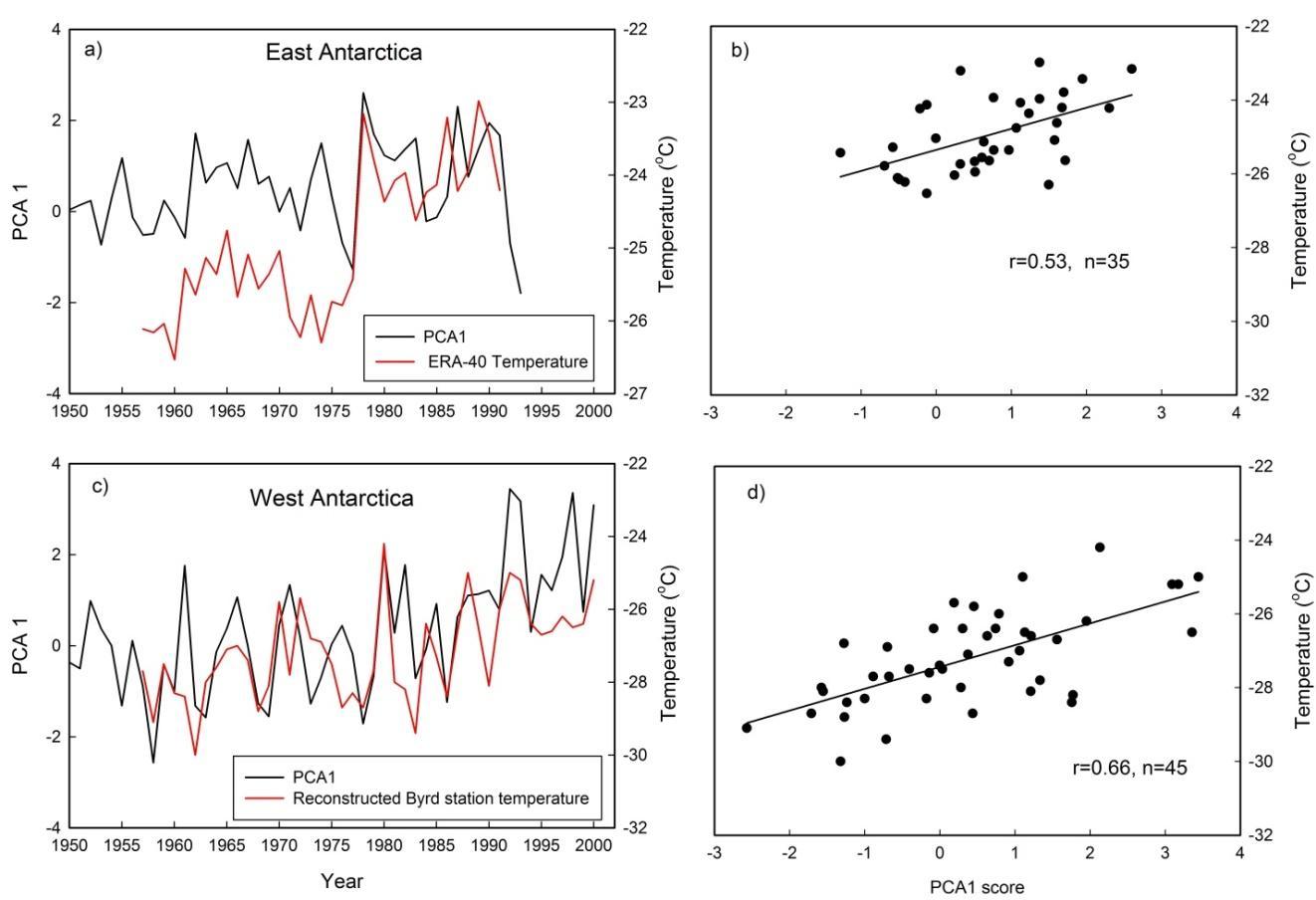


Fig. S2 Extraction of temperature signals from the oxygen isotopes records of multiple ice cores: a, b) First principal component (PC1) of the five ice core records of δ^18^O from east and the west Antarctic sector are plotted. The PC1 of east Antarctic and west Antarctica are compared with the temperature records from ERA 40 Reanalysis and Byrd station data (<http://polarmet.osu.edu/datasets/Byrd_recon/>). (c, d) PC1 of the east and west Antarctica are plotted with these temperature records show significant correlation; East Antarctica (p<0.05, r=0.53, n=35) and west Antarctica (p<0.05, r=0.66, n=45).

`
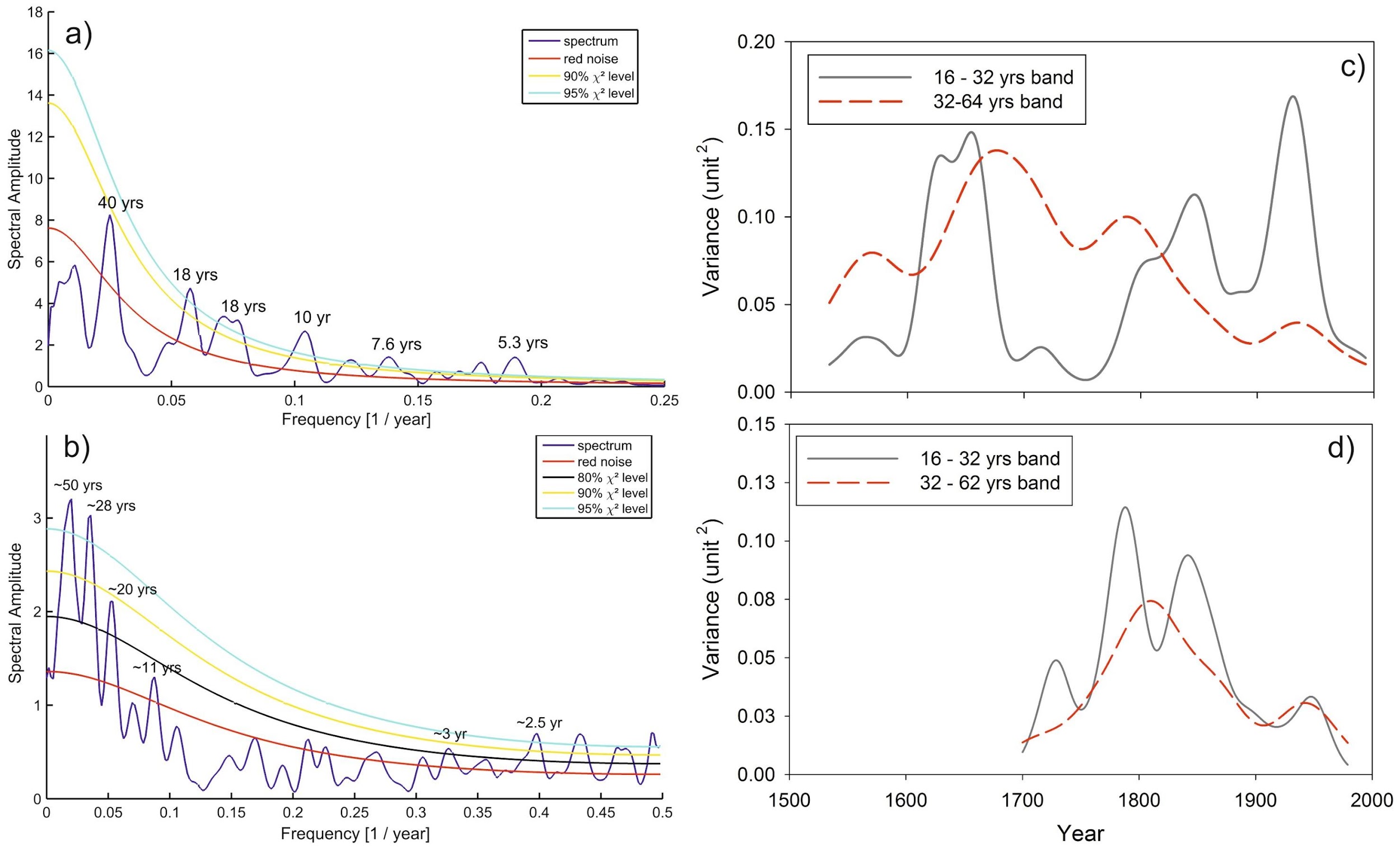


Fig. S3 a, b) Spectrum analysis of the reconstructed temperature of East Antarctica and PDO index of D'Arrigo^[12](#_ENREF_12)^ using REDFIT software[^13^](#_ENREF_13). Both of them show higher amplitude significant (90% χ^2^ level) periodicities at 16 – 32 years and 32 – 64 years band. c, d) Scaled average variance of east Antarctic temperature and PDO index of D'Arrigo^[12](#_ENREF_12)^ at 16 – 32 and 32 – 64 year band plotted with years.

Table-S2 Pearson correlation of East Antarctic temperature with Datwyler SAM index[^14^](#_ENREF_14) at different time interval.

|  |  |  |  |
| --- | --- | --- | --- |
| **Correlation period** | **p value** | **n** | **Correlation (r)** |
| 1900-1991 CE | p<0.001 | 91 | 0.487 |
| 1850-1991 CE | p<0.001 | 141 | 0.433 |
| 1533-1850 CE | 0.2096 | 317 | 0.075 |
| 1533-1991 CE | 0.0112 | 458 | 0.177 |
|  |  |  |  |


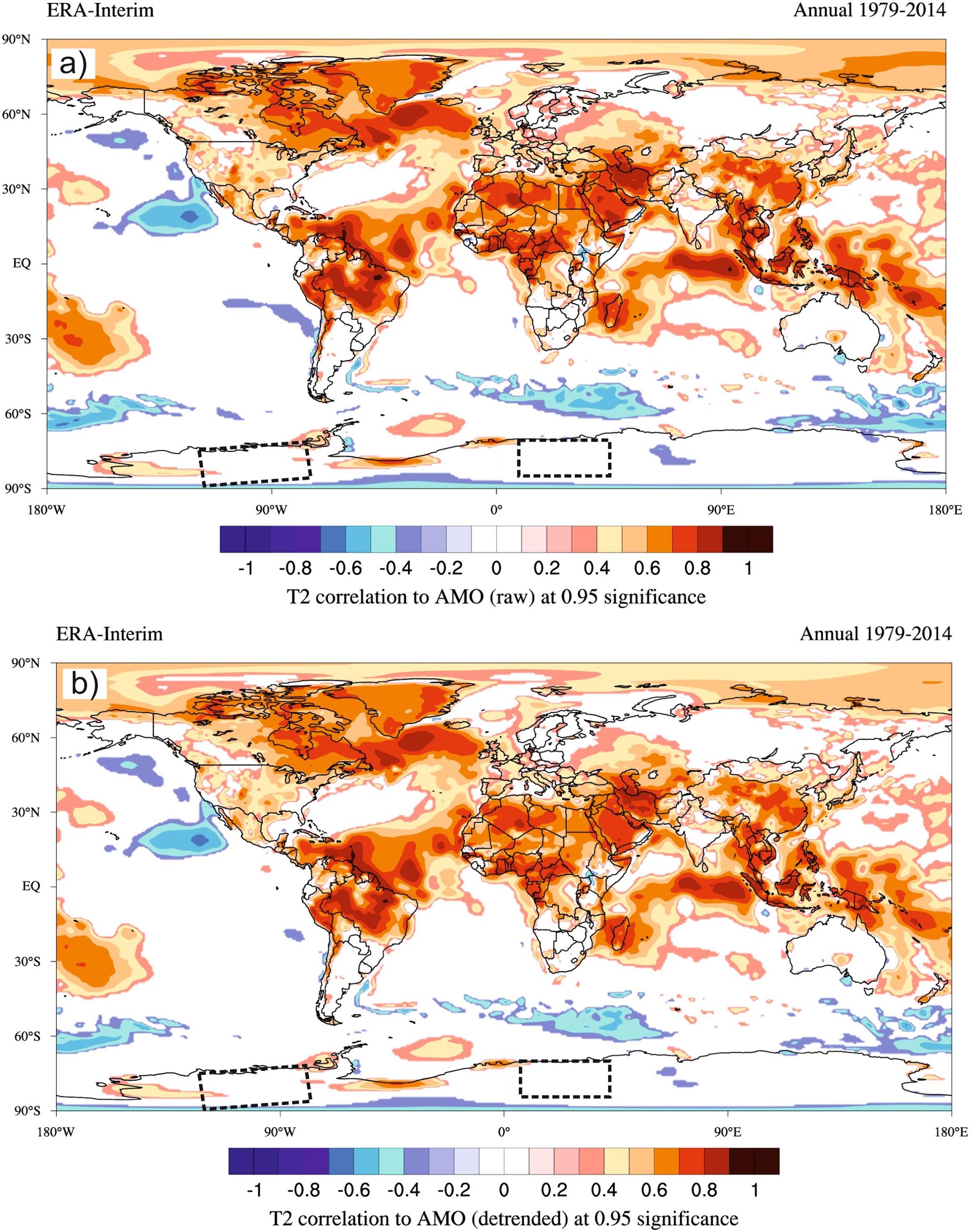


Fig. S4 Spatial correlations of ERA-Interim Reanalysis mean annual temperature with Atlantic Multi-decadal Oscillations (AMO) index. Regions of significant correlation (at 90% significance level) are highlighted in color band. Rectangles with dashed line represent core locations in East and West Antarctica. This plot was generated using Climate Reanalyzer (http://cci-reanalyzer.org), Climate Change Institute, University of Maine, USA.


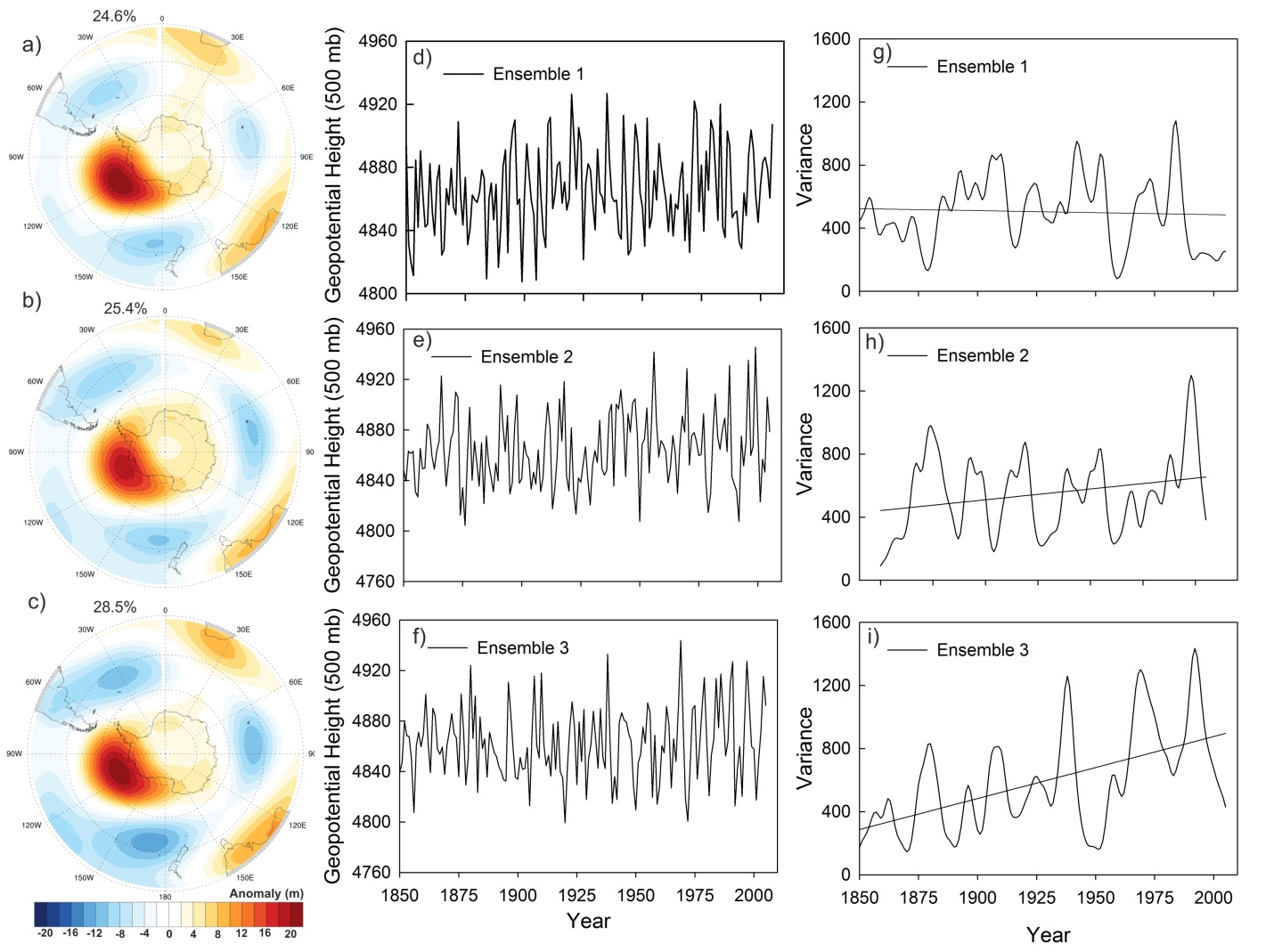


Fig. S5 (a, b, c) First mode of Empirical orthogonal functions (EOF) from Sept-Oct-Nov averaged 500mb geopotential height (z500) (m) over the southern hemisphere (30° S - 90° S; 0 - 360° E) for the period 1850-2005 from three ensemble members from the greenhouse gas forced simulations in CMSE-LME project. This plot was generated using the NCAR Command Language (Version 6.3.0) [Software]. (2015), Boulder, Colorado: UCAR/NCAR/CISL/TDD. http://dx.doi.org/10.5065/D6WD3XH5. The numbers in the left top corner indicates the percentage of variance explained by corresponding EOF pattern. (d, e, f) The middle panel shows the Sept-Oct-Nov average z500 (m) computed over the region 60°S - 75°S, 60°W – 120°W and (g, h, i) corresponding average variance at 2-8 year band are shown in right panel.

**
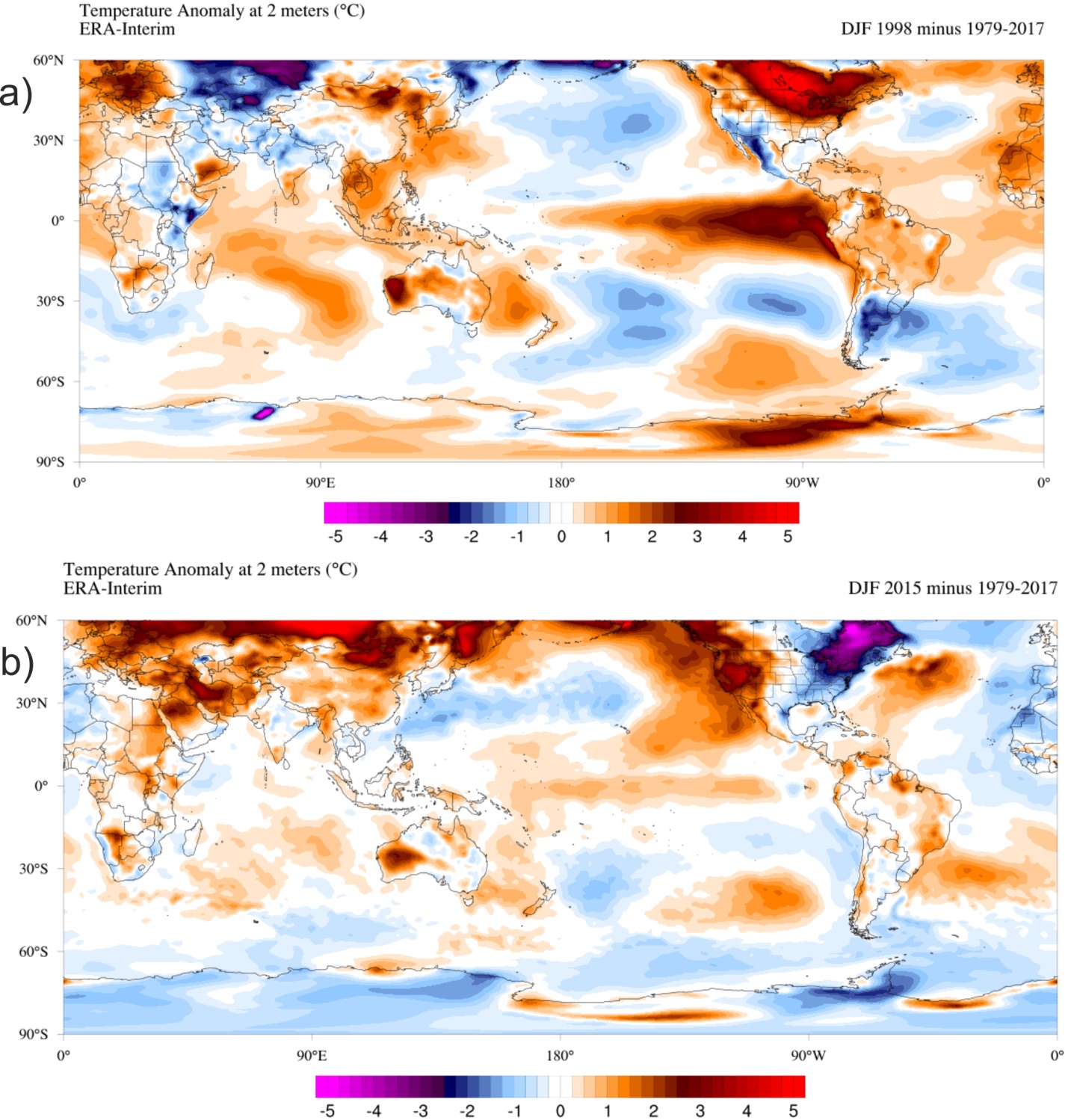
**

Fig. S6 Longitudinal changes in SST anomaly during (a) EP type El-Niño (1997/98, Dec-Feb) and CP type El-Nino (2015/16, Dec-Feb). This clearly shows that surface temperature distribution in Antarctica during the EP and CP El-Niño are different. This plot was generated using the NCAR Command Language (Version 6.3.0) [Software]. (2015), Boulder, Colorado: UCAR/NCAR/CISL/TDD. http://dx.doi.org/10.5065/D6WD3XH5.

**References**

1 Consortium, P. A. k. A global multiproxy database for temperature reconstructions of the Common Era. *Scientific Data* **4**, 170088, doi:10.1038/sdata.2017.88

https://[www.nature.com/articles/sdata201788#supplementary-information](http://www.nature.com/articles/sdata201788#supplementary-information) (2017).

2 Sommer, S., Wagenbach, D., Mulvaney, R. & Fischer, H. Glacio-chemical study spanning the past 2 kyr on three ice cores from Dronning Maud Land, Antarctica: 2. Seasonally resolved chemical records. *Journal of Geophysical Research: Atmospheres* **105**, 29423-29433, doi:10.1029/2000JD900450 (2000).

3 Thamban, M., Naik, S. S., Laluraj, C. M., Chaturvedi, A. & Ravindra, R. in *Earth System Processes and Disaster Management* (eds Rajiv Sinha & Rasik Ravindra) 51-66 (Springer Berlin Heidelberg, 2013).

4 Laluraj, C. M. *et al.* Origin and characterisation of microparticles in an ice core from the Central Dronning Maud Land, East Antarctica. *Environmental Monitoring and Assessment* **149**, 377-383, doi:10.1007/s10661-008-0212-y (2009).

5 Schneider, D. P. & Steig, E. J. Ice cores record significant 1940s Antarctic warmth related to tropical climate variability. *Proceedings of the National Academy of Sciences* **105**, 12154-12158, doi:10.1073/pnas.0803627105 (2008).

6 Steig, E. J. *et al.* High-resolution ice cores from US ITASE (West Antarctica): development and validation of chronologies and determination of precision and accuracy. *Annals of Glaciology* **41**, 77-84, doi:10.3189/172756405781813311 (2017).

7 Mitchell, L. E., Brook, E. J., Sowers, T., McConnell, J. R. & Taylor, K. Multidecadal variability of atmospheric methane, 1000–1800 C.E. *Journal of Geophysical Research: Biogeosciences* **116**, doi:doi:10.1029/2010JG001441 (2011).

8 Mosley-Thompson, E. *et al.* Glaciological studies at Siple Station (Antarctica): potential ice-core paleoclimatic record. *Journal of Glaciology* **37**, 11-22, doi:10.3189/S002214300004274X (2017).

9 Neftel, A., Moor, E., Oeschger, H. & Stauffer, B. Evidence from polar ice cores for the increase in atmospheric CO2 in the past two centuries. *Nature* **315**, 45, doi:10.1038/315045a0 (1985).

10 Thomas, E. R., Dennis, P. F., Bracegirdle, T. J. & Franzke, C. Ice core evidence for significant 100-year regional warming on the Antarctic Peninsula. *Geophysical Research Letters* **36**, L20704, doi:10.1029/2009GL040104 (2009).

11 Masson-Delmotte, V. *et al.* A Review of Antarctic Surface Snow Isotopic Composition: Observations, Atmospheric Circulation, and Isotopic Modeling. *Journal of Climate* **21**, 3359-3387, doi:10.1175/2007jcli2139.1 (2008).

12 D’Arrigo, R. *et al.* Tropical–North Pacific Climate Linkages over the Past Four Centuries. *Journal of Climate* **18**, 5253-5265, doi:10.1175/jcli3602.1 (2005).

13 Schulz, M. & Mudelsee, M. REDFIT: estimating red-noise spectra directly from unevenly spaced paleoclimatic time series. *Computers & Geosciences* **28**, 421-426, doi:https://doi.org/10.1016/S0098-3004(01)00044-9 (2002).

14 Dätwyler, C. *et al.* Teleconnection stationarity, variability and trends of the Southern Annular Mode (SAM) during the last millennium. *Climate Dynamics*, doi:10.1007/s00382-017-4015-0 (2017).
